# State-Dependent Memory: Neurobiological Advances and Prospects for Translation to Dissociative Amnesia

**DOI:** 10.3389/fnbeh.2018.00259

**Published:** 2018-10-31

**Authors:** Jelena Radulovic, Royce Lee, Andrew Ortony

**Affiliations:** ^1^Department of Psychiatry and Behavioral Sciences, Northwestern University, Chicago, IL, United States; ^2^Department of Psychiatry and Behavioral Neuroscience, The University of Chicago, Chicago, IL, United States; ^3^Department of Psychology, Northwestern University, Evanston, IL, United States

**Keywords:** dissociative amnesia, state-dependent memory, episodic memory, neuronal oscillations, neuronal connectivity, animal models, excitation/inhibition dynamics, stress

## Abstract

In susceptible individuals, overwhelming traumatic stress often results in severe abnormalities of memory processing, manifested either as the uncontrollable emergence of memories (flashbacks) or as an inability to remember events (dissociative amnesia, DA) that are usually, but not necessarily, related to the stressful experience. These memory abnormalities are often the source of debilitating psychopathologies such as anxiety, depression and social dysfunction. The question of why memory for some traumatic experiences is compromised while other comparably traumatic experiences are remembered perfectly well, both within and across individuals, has puzzled clinicians for decades. In this article, we present clinical, cognitive, and neurobiological perspectives on memory research relevant to DA. In particular, we examine the role of state dependent memory (wherein memories are difficult to recall unless the conditions at encoding and recall are similar), and discuss how advances in the neurobiology of state-dependent memory (SDM) gleaned from animal studies might be translated to humans.

## Introduction

Normal states of consciousness involve an ongoing awareness of oneself and one’s environment. Nevertheless, many everyday experiences such as daydreaming, losing track of time, being submerged in a play, a novel, or a movie, are manifestations of temporary dissociation from normal states of consciousness. Getting into, and especially getting out of these states is typically relatively easy, so they are usually considered to be normal. However, in some individuals, particularly those who have been exposed to psychological trauma, dissociation occurs unconsciously and cannot be controlled. In such pathological cases, dissociation is viewed as a “disruption of and/or discontinuity in the subjective integration of one or more aspects of psychological functioning, including—but not limited to—memory, identity, consciousness, perception and motor control” (Spiegel et al., 2011b). Some researchers believe that by compartmentalizing these psychobiological functions, trauma-related threats and distress can be separated from conscious awareness, preventing the experience of pain, discomfort and anxiety, and promoting coping and survival in the face of overwhelming traumatic stressors (Putnam, 1989; Herman, 1992). From this perspective, dissociation can be viewed as adaptive. However, dissociation is maladaptive when it persists and is used to cope with everyday stressors that do not pose a significant threat (Haugaard, 2004; Schimmenti and Caretti, 2016; Choi et al., 2017). Under these conditions, dissociation can disrupt the development of self-regulatory processes in stress response systems and can lead to the development of persistent self-dysregulation and dissociative disorders (Curtois and Ford, 2009).

Based on the most prevalent symptomatology, dissociative disorders include dissociative amnesia (DA, inability to access important autobiographical and other memories), dissociative identity disorder (fragmentation of identity and formation of multiple personalities), and depersonalization-derealization disorder (detachment from self or the environment; Spiegel et al., 2011a). DA, which used to be called memory repression, is a manifestation of dissociative disorders that predominantly affects memory systems and enables individuals to detach from the past. The earliest theoretical accounts of such memory failures proposed that overwhelming stress prevents the adequate integration of traumatic and normal conscious experiences (Janet, 1889). Importantly, it was also recognized that although the trauma-related memories were inaccessible their continued existence was manifested through affective and behavioral symptoms (Janet, 1889; Breuer and Freud, 1955). Newer accounts similarly define DA as a process whereby individuals automatically lose access to (dissociate from) memories of an entire traumatic event or details of that event, resulting in significant memory gaps or in no memory at all (Wolf and Nochajski, 2013). DA can also generalize to identity and life history, causing clinically significant distress or impairment in social, occupational, affective and other important areas of functioning. Consistent with the view that dissociation is maladaptive, peritraumatic dissociation is seen as a strong risk factor for the development of PTSD (Briere et al., 2005). However, in some cases DA can also be protective, as evidenced in survivors of childhood sexual abuse suffering from DA who had less depression and anxiety than survivors without it (Coifman et al., 2007).

While improving memory access in the case of generalized DA is an important therapeutic goal, accessing specific traumatic memories can have unpredictable consequences. For some patients, it delays recovery and worsens symptoms. For example, although survivors of childhood sexual abuse suffering from DA tend to suffer less from depression and anxiety, if their memories of the traumatic event surface, these individuals are at increased risk of experiencing higher levels of traumatic symptoms compared to survivors who have never experienced DA (Bonanno et al., 2003). Similarly, the successful recall of traumatic memories can sometimes be highly stressful and can cause symptoms of PTSD or suicidal urges (Fetkewicz et al., 2000). In contrast to these observations, in other individuals, regaining access to trauma-related memories results in positive outcomes because once the memories have been accessed, psychotherapy can help such individuals understand how trauma caused their amnesia, how it disrupted their lives, and how their issues can be resolved so as to help prevent further trauma-related symptoms in the future (Staniloiu and Markowitsch, 2014; Sharma et al., 2015; Cassel and Humphreys, 2016; Markowitsch and Staniloiu, 2016).

Several mechanisms have been proposed to explain restricted access to unwanted traumatic memories. Historically, the idea was that such memories are voluntarily repressed (Breuer and Freud, 1955)—a view that has recently been re-conceptualized as executive control of memory access (Anderson and Green, 2001; Anderson et al., 2004). An alternative view proposes an automatic process wherein such memories are state-dependent in that their accessibility is critically dependent on the congruence between the encoding and retrieval conditions (Putnam, 1989; Eich, 1995; Harrison et al., 2017). Although both mechanisms might be playing a role, here we will focus on the relationship between state-dependent memory (SDM) and DA because of recent progress in the understanding of the neurobiology of SDM. The remainder of this article is divided into four main sections. First, we present a case study of an individual, which provides a vivid example of DA (“A Case Study” Section). We then, in Section “SDM as a Gateway to DA—the Human Cognitive Perspective”, move on to a discussion of the cognitive foundations of SDM and, especially, of episodic memory, and lay out their relation to DA. Findings from animal and human research emphasizing neurobiological aspects of SDM are reviewed in Section Memory and SDM—a Neurobiological Perspective. In Section Implications of Neurobiological Research for Human DA”, we return to the human level and present a brief discussion of the implications of our current understanding of current neurobiological knowledge for DA, after which, in Section “Revisiting Skepticism Concerning DA”, we conclude with a discussion of some standard objections to the concept of DA itself.

## A Case Study

The complexity of stress-related disorders can best be illustrated by an example of a clinical case that demonstrates the impact of psychological trauma. The case we have chosen illustrates several common features of DA including high comorbidity with other mental disorders, an inability to recall a life-threatening traumatic memory that was readily recalled by family members, the occurrence of flashbacks after withdrawal from a hypnotic drug (clonazepam) treatment, and full recovery of the memory accompanied by significant clinical improvement following prolonged exposure (PE) psychotherapy.

“Patient *X* is a 60-year-old male who presented with new onset symptoms consistent with PTSD with dissociative symptoms, delayed expression (309.81 (F43.10)). The index trauma was a house fire 15 years ago, in which he evacuated his granddaughter and sister-in-law while suffering from smoke inhalation. Symptoms included nightmares of the fire, recurrent and involuntary memories of the event, dissociative symptoms including flashbacks, DA in the form of an inability to readily recall details about the fire, avoidance of talking about the fire, profound guilt, severe insomnia and hyperarousal. Of interest, the re-experiencing symptoms did not begin until the present time, a decade and a half after the fire, although hyperarousal symptoms have been chronic. The recent *onset of nightmares, re-experiencing and dissociative flashbacks was temporally correlated with a reduction and elimination of clonazepam*, used to treat a longstanding severe insomnia that was resistant to Cognitive Behavioral Therapy (CBT) for insomnia. His medical history is significant for a history of childhood epilepsy and remote, pre-trauma history of left middle cerebral artery with sparing of the medial temporal lobes but damage to the left parietal lobes. His Montreal Cognitive Assessment (MoCA) score is 29, with no deficits in declarative or procedural memory, but a mild dysarthria. There is an additional adult history of psychogenic non-epileptic seizures, confirmed by inpatient video EEG recordings of grand mal and partial seizures without correlated EEG activity. He has been on long term anti-epileptic treatment that has remained stable through the period from the fire to the present, including phenobarbital 120 mg qHS and gabapentin 600 mg TID. His psychiatric history is extensive, with a diagnosis of borderline personality disorder since adolescence, with multiple inpatient psychiatric hospitalizations for suicidal ideation and at least three suicide attempts.

Chart review reveals prominent DA regarding the fire. About 1 week after the fire, he was hospitalized at two different hospitals, for nearly 2 months for intractable seizures. It was only during the second hospitalization that the diagnosis of psychogenic non-epileptic seizures was made. Extraordinarily, *no mention was made during this 2-month period of the fire by the patient nor the medical chart*, despite psychiatric consultations for suicidality. The patient retrospectively reports that from the beginning of the neurological hospitalization to the present day, he has not thought about the fire and has not discussed it with any of his health providers. His subjective experience of that time is of “waking up” in the hospital after a period of sedation in a state of shock, but *without memory, or awareness of memory, of the fire*. Interestingly, the family corroborates the account of the fire by the patient. His PTSD Checklist-Civilian Version (PCL-C) score was 64, exceeding proposed thresholds for clinically significant PTSD severity. A course of weekly PE psychotherapy was initiated. PE is an evidenced based treatment for PTSD with established efficacy (reviewed in Foa, 2006). During initial imaginal exposure sessions, the level of fear-related arousal was relatively low. The predominant source of distress was shame at not being able to prevent the fire. After six sessions, *during imaginal exposure, the levels of distress related to fear increased, to subjectively maximal levels of intensity (100% of experienced fear intensity)*. Simultaneously, the PCL-C scores began to decrease to 55. During session seven, he had a short seizure during imaginal exposure, coinciding with peak subjectively experienced fear. The seizure was consistent with previous psychogenic non-epileptic seizures. During session eight, he revealed that in the previous week, practicing imaginal exposure at home resulted in psychogenic non-epileptic seizures about 50% of time with prolonged periods of dissociative state. In session eight, session imaginal exposure was modified to be conducted with eyes open rather than closed. Additionally, when dissociative symptoms started, verbal reorienting was provided, followed by resumption of imaginal exposure. He was to practice imaginal exposure at home with his lapdog present, who has been trained to lick the patients face at signs of dissociation or seizure. At session nine, the PCL-C score for the previous week decreased further to 41, with a marked reduction in frequency of nightmares. He was able to complete PE over the course of six more sessions, without a return of seizures during session, and a final PCL-C score of 21.

In summary, this was case of delayed onset of PTSD, with onset of symptoms occurring a decade and a half after the trauma. In the interim, there was documented evidence of DA, which is relatively common in patients suffering from borderline personality disorder (Sar et al., 2014). Family members confirmed the severity of the fire and involvement of the patient. The trigger for the onset of PTSD symptoms was the elimination of a nighttime dose of clonazepam. During a course of PE, there was further reduction of DA, specifically of emotional numbing, which had previously blocked access to the subjective experience of fear of dying due to smoke inhalation and heat exposure. Although access to intense feelings of fear and distress when retrieving of details of the traumatic experience led to transient states of dissociation and even non-epileptic seizures during a session, continuing PE therapy resulted in habituation and reduction of fear and distress. The dissociative states and non-epileptic seizures did not return.

Even though in some cases, as in this one, therapy seems to be successful, in general there is disappointingly little evidence-based research to inform successful approaches to the treatment of DA. This might be due in part to a bitter controversy in the field that arose in the 1990’s as to whether DA is a real phenomenon. The controversy, which came to be known as the “Memory Wars” (after the widely publicized book by Crews, 1995), was largely a reaction to psychodynamic approaches to DA (in particular those arising from cases of alleged childhood sexual abuse). Issues of central concern related to “repression” as a specific mechanism of memory inaccessibility (Breuer and Freud, 1955), as well as to the problem of distinguishing false from veridical memories (Loftus and Davis, 2006) and dissociated from non-dissociated memories (McNally, 2007). Fortunately, advances in both human and animal research in the neurobiology of memory are providing new insights in light of which many such questions can be newly addressed.

## State-Dependent Memory as a Gateway to DA—The Human Cognitive Perspective

In seminal work on the relationship between stress and memory, Brewin et al. (1996) proposed that traumatic experiences give rise to two types of memory representations. One type results from the conscious processing of the trauma and includes accessible memories that can be expressed verbally, while the other results from the unconscious processing of the trauma. Brewin et al. (1996) referred to the result of this latter type of memorial representation as “situationally accessible knowledge,” which they argued is automatically retrieved when a person is in a situation that is similar to the one in which the trauma was experienced, a view supported by many others (Eich, 1995; van der Kolk and Fisler, 1995; Whitfield, 1995). This account of “situationally accessible knowledge” fits well the definition of the phenomenon of SDM that we discuss in depth below. However, before doing so, we need to set the stage by briefly reviewing some key concepts in human memory.

### Memory Systems

Understanding the basic issues relating to memory ought to be simple: through experience and learning, we *acquire information*, we *encode* it, *retain* it for later use, and when we need it, we *retrieve* it. But, of course, it isn’t that simple, in part because memory is not a unitary concept and such a bare-bones account inevitably neglects its rich complexity. Cognitive psychologists have identified all manner of different kinds of memory—iconic, haptic, echoic, short-term, working, long-term, declarative, non-declarative, procedural, semantic, episodic, implicit, explicit and more. These different kinds of memory, or memory systems, can be thought of as (collections of) different kinds of specific memories, and they are distinguished in terms of the nature of their content, their durability, and the way in which they are acquired and accessed.

The most relevant top-level aspect of the human memory system is long-term memory, which comprises information that is retained for a long time—days, weeks, months, or years, rather than seconds, minutes, or hours. Long-term memory is comprised of non-declarative and declarative memory (Squire, 1992), a distinction which is reminiscent of the classic partitioning of knowledge into *knowing how* and *knowing that* (Ryle, 1945). The brain has the capacity to store vast amounts of information that is used to organize behavior and make decisions, and much of this information is part of the non-declarative memory system, which means that it can be accessed and used automatically without the need to voluntarily retrieve it. Non-declarative memory includes procedural memory (or knowledge) such as one’s knowledge of how to ride a bicycle, as well as the results of simple classical conditioning, of perceptual learning, and of non associative learning (e.g., habituation).

By contrast, declarative memories are records of specific facts and events that can normally be intentionally recalled. Memories of facts and events can be talked about, they can be articulated in language, they can be reported; hence, “declarative.” The declarative memory system consists of episodic memory, with which this article is primarily concerned, and semantic memory. Individual episodic memories are representations of actual experiences that generally incorporate the spatial, sensory, and temporal information associated with those experiences, integrated into a unitary whole. In its original formulation (Tulving, 1972), the episodic memory system was characterized as the totality of a person’s encoded personal experiences—an autobiographical record of experienced events and their temporal and spatial contexts. Subsequently, Tulving (1985) modified the idea, tying it more explicitly to the conscious act of remembering a past experience. On the revised view, for something to count as an episodic memory it was not sufficient that the remember merely know (or believe) that something happened to him or her. That kind of factual knowledge, even though it is knowledge about the self, is better thought of as belonging to semantic memory. Rather, Tulving proposed that the construct of episodic memory capture the awareness—the autonoetic consciousness—associated with the actual act of remembering, for it is this that bestows the “special phenomenal flavor to the remembering of past events” (p. 3). On this view, what matters is the *remembering of the experience itself* rather than the remembering of the fact of the experience (Markowitsch and Staniloiu, 2016).

### The (in)fidelity of Episodic Memories

An important aspect of individual memories is their degree of fidelity. Fidelity has to do with the relation between what was experienced and what was encoded, and between what was encoded and what was retrieved, and thus concerns the integrity of the information encoded or retrieved. It is well-established that memories of meaningful information are rarely exact records of what was seen or heard (Bartlett, 1932; Bransford and Franks, 1971; Anderson and Ortony, 1975). In general, there is very little information that is encoded and retained verbatim. Instead, even at the time of encoding, what is encountered routinely contains omitted as well as elaborated and even intruded information, often the result of unconscious inferences. Thus, what is encoded is not the raw sensory or semantic input, but a representation constructed from that input. Furthermore, just as encoding is a constructive process, so is retrieval. The most celebrated early exponent of this (re)constructive view of memory is Frederick Bartlett who undertook detailed experimental work on memory for drawings and stories. Bartlett’s work, and that of many after him, established conclusively that memory for meaningful material normally involves the unconscious elaboration of stored fragments of that material enhanced with general world knowledge, associations and conventional ideas and schemas. In addition, memory for such material is also influenced by subsequent exposure to relevant related information as well as by subsequent successful or unsuccessful attempts to recall it.

The basic principles relating to the constructive nature of memory encoding and retrieval mean that the fidelity of the relation between what was encountered and what is encoded can in no way be guaranteed, nor can the fidelity of the relation between what was encoded and what is or can be retrieved. Thus, even under normal conditions, memory distortions, memory failures, and even false memories are routine psychological phenomena.

### Sources of Problems in Accessing Episodic Memories

The concepts of *retrieval* and *forgetting* are, of course, central to any discussion of memory and memory-related disorders. Assuming that forgetting is some sort of failure of retrieval, we can start by asking what retrieval is. One might think that retrieval is simply the process of recovering or locating information stored in memory (VandenBos, 2015). However, this kind of definition is too course-grained to be useful, not least because it fails to acknowledge two crucially different processes, namely, *intentional* vs. *unintentional access* to stored information—the willful effort to retrieve, also called recall, vs. the incidental, unintended activation of such information, as happens in many cases of recognition and reminding where, material comes to mind unbidden. In our discussion of memory processes in humans, we shall primarily be concerned with the intentional process of *retrieval* rather than with unintentional processes, and with the nature and consequences of failures of retrieval (i.e., the forgetting) of episodic memories.

Forgetting can occur for one of two general reasons: either the to-be-remembered material itself is compromised, or access to that material is compromised. When the memory itself is compromised, forgetting can occur because its contents have degraded so that only fragments of the original memory remain, or in some cases because the memory is degraded to such an extent that there is nothing coherent to access at all. But, forgetting can also result from a failure of the retrieval mechanism to access an intact memory. In such cases, access to the to-be-recalled representation is for one reason or another, temporarily (or even permanently) blocked, as in the case of patient X, described in the case study, who for 15 years was unable to retrieve his traumatic involvement in a frightening house fire.

Of particular interest in the present context is the kind of retrieval failure that occurs when the conditions at time of retrieval differ from those that pertained at the time of encoding. Although known as SDM, this phenomenon might be thought of as a special case of blocked-access forgetting, because state-dependency could be a feature of the access mechanism rather than (or in addition to) a feature of the memory itself. This kind of state-dependent forgetting is particularly important because of its potential relevance to DA. We will therefore now discuss it in greater detail, although referring to it by its more conventional name of SDM.

Formal definitions of SDM hone in on the psychological, biological, or physical states of the rememberer. For example, dictionaries of psychology define SDM as “the tendency for information that was learnt in a particular *mental or physical state* to be most easily remembered in a similar state” (Colman, 2009 italics added), or as “a condition in which memory for a past event is improved when the person is in the same *biological or psychological state* as when the memory was initially formed” (VandenBos, 2015, italics added). State dependence is a quite general phenomenon which to some degree is characteristic of many kinds of memories. An interesting anecdotal example of its relevance to cases other than episodic memory, and which is also of some historical interest, is mentioned by Godden and Baddeley (1975). They noted that John Locke, the 17th century British philosopher, wrote of a man who, having learned to dance “to great perfection” in a room in which there was a wooden trunk, could then only perform well what he had learned in that or a similar room, and one in which was situated a similarly placed trunk. This would be an example of state-dependent procedural memory. State-dependency can also arise in simple conditioning. Indeed, the first documented experiment demonstrating SDM (Girden and Culler, 1937) was in the context of a conditioned reflex in dogs, with learning taking place under conditions quite different from normal states of consciousness. These authors showed that a conditioned reflex induced under curare could not be induced at all when the dogs were awake, there being a complete amnestic barrier between the normal and curare-induced states.

Even though it is important to recognize that state dependence is a phenomenon that occurs in the context of other kinds of memory, its occurrence in the context of episodic memories is particularly interesting because episodic memories, reflecting as they do personal experiences, are by definition the kind of memories that can be consciously recalled. In their classic experiment, Godden and Baddeley (1975), referring to their particular case of SDM as context-dependent memory, demonstrated that the recall of word lists that scuba divers had learned under water was superior when the divers were again under water than when they were on dry land. The phenomenon has also been demonstrated when acquisition of the to-be-remembered information occurred under the influence of psychoactive drugs such as alcohol (Weingartner et al., 1976) and marihuana (Hill et al., 1973); and essentially the same phenomenon, but under the label of *encoding specificity* (Thomson and Tulving, 1970; Tulving and Thomson, 1973) was demonstrated by showing that retrieval of items from episodic memory was optimal when the conditions at the time of retrieval, such as context or available cues, were the same as those at the time of encoding.

It should be noted that the fact that state-dependency is about the optimal conditions for accessing items stored in episodic memory does not mean that absent those conditions, memory access will necessarily fail. In fact, state-dependence is perhaps best be thought of as a variable that can affect the ease of access, ranging from minimal if any influence at one end of the continuum to substantial influence at the other. It might be that cases of SDM in which access to a memory is highly restricted, representing the extreme (high-influence) end of the continuum, are qualitatively different from other cases. These would be the cases of most relevance for DA.

## Memory and SDM—A Neurobiological Perspective

DA, flashbacks and other dissociative phenomena have frequently been observed not only as a result of traumatic stress, but also as a result of the use of dissociative drugs such as PCP, ketamine, or LSD (Brna and Wilson, 1990). However, due to ethical and regulatory issues, such drugs cannot be used in human SDM research. For this reason, most of our current knowledge on the neurobiology of SDM is based on animal studies. Using tools for visualizing and manipulating neurons directly involved in memory processing in animals (Gradinaru et al., 2010; Zhu and Roth, 2014), it is now possible to study the memory circuits that process veridical memories, false memories and SDMs (Garner et al., 2012; Ramirez et al., 2013; Liu et al., 2014; Jovasevic et al., 2015). We are thus in an unprecedented position to explore the connection between state-dependency and memory access in DA. In fact, animal models allow us to examine extreme cases of SDM, in which memories cannot be retrieved at all under normal conditions (“complete amnestic barrier” Overton, 1991). Below, we discuss the strengths and limitations of animal approaches to episodic-like memories including SDM, highlighting the relevance of emerging findings to our understanding of DA and possibly other memory-related psychopathologies.

### Modeling Episodic-Like Memories and SDM in Rodents

Robust memories of stressful experiences that persist over months or years (Gale et al., 2004) can be readily induced in experimental animals. This is typically done using contextual fear conditioning or passive avoidance learning in which animals—most often, rodents—learn to associate multisensory environmental contexts with aversive foot shocks. As evidence that such learning has occurred, upon re-exposure to the conditioning context, rodents express either freezing behavior (contextual fear conditioning if the animals cannot escape) or avoidance behavior (passive avoidance if they can escape). Such memories resemble human episodic memories in that they require the integration of spatial, multisensory, and temporal information into memory, and this integrated representation has to be accessed for freezing or avoidance behavior to occur (Fanselow, 1990; McGaugh and Roozendaal, 2002). Both in rodents and humans, these memories depend on neuroanatomical mechanisms which differ from those required for conditioning to simple cues (Kim and Fanselow, 1992; Phillips and LeDoux, 1992). The only aspect of human episodic memory that we cannot test in animal models is the subjective re-experiencing of the encoded event, for which reason we use the expression *episodic-like memory* when discussing animal research. Despite this limitation, the neurobiological basis of the processing of episodic-like memories in animals is known to be surprisingly similar to that in humans (Grillon, 2002; Milad et al., 2006; Rauch et al., 2006) and can thus be successfully used to develop mechanistic hypotheses related to memory processes.

From a neurobiological point of view, memories are most likely encoded and retrieved as sequences of neuronal activity (Eichenbaum, 2000; Hasselmo, 2005; Pastalkova et al., 2008; Carr and Frank, 2012) bound and ordered by neuronal oscillations (Lisman and Jensen, 2013). These patterns of activity vary with neuronal connectivity and other properties, such as strength of synaptic connections and responsiveness to excitatory and inhibitory input, which are determined by the genetic and molecular profile of each neuron. These features change in different brain states, sometimes resulting in state-dependent encoding of memories, as reviewed recently (Radulovic, 2017; Radulovic et al., 2017). Furthermore, it appears that even initially accessible memories can be rendered state-dependent if the brain states are manipulated at the time of retrieval (Sierra et al., 2013; Gisquet-Verrier et al., 2015). Such memories are no longer accessible under the conditions present at encoding, but instead, under the conditions present at retrieval. Thus, there is much evidence that the efficient encoding of memories, but with only limited subsequent access, is possible.

### Mechanisms of SDM: the Role of Excitatory/Inhibitory Balance and Stress

The standard approach to studying SDM is to manipulate the brain states of animals by using various drugs (Netto et al., 1987; Overton, 1991; Colpaert et al., 2001; Rezayof et al., 2003). Such pharmacological approaches have unique advantages such as allowing for rigorous control over the experimental conditions (e.g., dose, injection site, memory phase), and enabling the determination of the role of individual neurotransmitter systems. This means that we can investigate mechanisms that causally contribute to memory processing in different brain states and characterize these states at different scales of neuronal function. Moreover, animal models can be easily designed to test extreme cases of SDM that completely restrict memory access, which could be particularly relevant to DA.

At the level of neurotransmission, the encoding and retrieval of episodic memories depend on the dynamics between neuronal excitation (mediated by glutamate) and inhibition (mediated by GABA; Froemke, 2015). Whereas excitatory neuronal networks are believed to play a key role in memory encoding and retrieval, inhibitory networks have been typically viewed as memory impairing (Rudolph and Möhler, 2006). However, this stance has been challenged by recent evidence indicating that while inhibitory networks do make memories quiescent, those memories nevertheless remain available for activation under particular conditions (Barron et al., 2016, 2017). Consistent with this view, many drugs used to generate SDM in animal models increase the inhibitory tone in the hippocampus (Radulovic et al., 2017). SDM can also be seen at the other extreme, namely under conditions of enhanced excitatory transmission, for example in response to psychostimulants and noradrenaline (Overton, 1991; Berridge and Waterhouse, 2003).

Just as drugs alter the local or global excitatory/inhibitory dynamics, so too do stressful experiences. In some cases, acute stress predominantly triggers release of glutamate (Popoli et al., 2011). In other stress paradigms, there are important individual variations, with some animals responding with low GABA/glutamate release (indicating relatively high excitation vs. inhibition) and others with high GABA/glutamate release (indicating relatively high inhibition vs. excitation) in the prefrontal cortex (Drouet et al., 2015). High inhibition has also been found after chronic stress in both adult (McKlveen et al., 2016) and juvenile (Albrecht et al., 2016) rodents. Notably, the effects of juvenile stress persisted throughout adulthood in a population of hippocampal neurons (dentate granule cells). These findings suggest that under some circumstances, and when stress is particularly severe (Drouet et al., 2015), the excitatory/inhibitory balance can shift towards inhibition or excessive excitation, both of which are more likely to result in SDMs than in easily accessible memories. A shift in excitatory/inhibitory balance in the direction of increased excitation could underlie the finding in patient X described in the case study, whose loss of memory of the fire was alleviated during clonazepam withdrawal, which typically results in overexcitation.

Although the similarities between stress-induced and drug-induced SDM are suggestive, it is important to note that reliable models of stress-induced SDM have yet to be developed and validated. To date, the field has mainly focused on stress-enhanced and extinction-resistant memories (Rau et al., 2005; Tronson et al., 2010) rather than inaccessible memories. Given the advances in our understanding of memory impairing effects of stress (Todorovic et al., 2007; Maras et al., 2014; Moreira et al., 2016), studying inaccessible memories is now feasible. The development of reliable animal models of stress-induced SDM is an important future challenge for the identification of a translational link between fundamental mechanisms identified in animals and psychopathologies in humans.

### Brain States That Subserve SDM

At first glance, the finding that susceptibility to SDM processing increases with the excitation/inhibition balance might suggest that SDM is a quantitative rather than a qualitative phenomenon. However, analyses at the level of network activity and connectivity indicate otherwise. Changes in the hippocampus, which is known to play important roles in processing both conscious and unconscious memories (Henke, 2010; Hannula and Greene, 2012; Shohamy and Turk-Browne, 2013), may be particularly relevant. A notable change within the hippocampus and cortex during SDM encoding under heightened tonic inhibition (using systemic infusion of the drug gaboxadol) is the increase in power of slow, delta oscillations, along with a decrease of theta and gamma oscillations (Meyer et al., 2017). At the same time, at the circuit level, the connectivity between the hippocampus and neocortical areas (retrosplenial, entorhinal and anterior cingulate cortex) is significantly reduced (Jovasevic et al., 2015; Meyer et al., 2017). Consistent with these observations, it was recently found that enhanced cortical delta oscillations causally contribute to the formation of state-dependent fear-inducing context memories during states of reduced excitation (using systemic administration of the NMDA receptor antagonist MK-801; Jiang et al., 2018).

Changes of network activity and connectivity have at least three important implications. First, they are consistent with the suggestion of Jacobs and Nadel (1998) that traumatic levels of stress lead to disconnections between memory processing brain areas. This view has been supported by recent imaging studies in patients with DA (Staniloiu and Markowitsch, 2012; Harrison et al., 2017; Thomas-Antérion, 2017). For example, patients show alterations of functional MRI imaging signals in frontal and temporal lobes (Hennig-Fast et al., 2008) with increased prefrontal and decreased hippocampal metabolic activity during testing for memory recall (Kikuchi et al., 2010). After treatment for DA, the pattern of brain activation normalized in a patient whose memories were recovered, whereas it remained unchanged in the patient whose memories were not recovered. Although these initial findings need to be confirmed in larger cohorts, they suggest a direct relationship between alterations of hippocampal and cortical activity and DA. Thus, as in other cases of memory inhibition (Anderson et al., 2004; Benoit et al., 2015), DA is often accompanied by increased activation in the dorso- and ventral-lateral prefrontal cortex, with associated deactivation in medial temporal structures, such as the hippocampus.

Second, the role of slow oscillations in SDM could explain an apparently paradoxical observation, namely, that access to traumatic memories can be facilitated not only under conditions of elevated stress, as seen in patient X during PE therapy, but also during therapies carried out while in hypnotic or relaxed states (Li et al., 2017). We know that the power of delta waves increases both during elevated arousal associated with severe stress (Kolassa et al., 2007; Ahnaou and Drinkenburg, 2016; Marshall and Cooper, 2017) and during states of sleep and relaxation (Knyazev, 2012). For example, a study of memories acquired under severe stress (near death experiences) found that the power of delta oscillations was positively correlated with memory details recalled during hypnosis, particularly with regard to the resolution, reliving, and spatiotemporal organization aspects of those memories (Palmieri et al., 2014).

Third, the findings pertaining to the relationship between delta oscillations and SDM could further our understanding of the relationship between brain oscillations and memory processes more generally. Both empirical data and computational modeling suggest that at the level of the hippocampus, memories are encoded as sequences (patterns) of neuronal activity that are combined into “chunks” of memories at the level of the cortex (Levy and Wu, 1996; Kesner and Rolls, 2015). Importantly, the size of these sequences depends on the balance between excitatory and inhibitory synaptic connections (Levy and Wu, 1996). Typically, theta oscillations are highly effective in binding components of episodic memories, however, this does not seem to be the case in human DA or extreme cases of SDM in animals. It is possible that traumatic memories and SDMs are particularly fragmented (van der Kolk and Fisler, 1995; Nadel and Jacobs, 1998) in which case slower delta oscillations might bind them more effectively than theta oscillations. However, this speculation would need to be tested experimentally.

All in all, neuroscience research in rodents demonstrates that depending on the conditions, stress-related experiences can be encoded either as robust memories or as impaired memories. According to our model, tress-related SDMs, as an example of the latter, would be particularly favored when the excitation/inhibition balance is shifted towards extremes, resulting in qualitatively different brain states in terms of brain oscillations and overall connectivity between hippocampal and cortical circuits, as illustrated in our recent work (Radulovic et al., 2017). We suggest that such states are likely to lead to the encoding of memories in more fragmented sequences that cannot be bound with high-frequency oscillations and therefore cannot be easily integrated in the hippocampal-cortical episodic memory circuits.

## Implications of Neurobiological Research for Human DA

Although the phenomenon of SDM has been recognized for a long time, our understanding of its underlying mechanisms is only in its early stages. Nevertheless, with advances in human neuroscience research, several findings from animal research can already be tested, and possibly translated to humans. The increasing sophistication of imaging techniques has paved the way for delineating the processing of real, imagined, and high-stress-induced memories (Palmieri et al., 2014). For example, by using dynamic causal models derived from data from EEG and fMRI studies (Legon et al., 2016), it is now possible to explore in great detail the excitatory/inhibitory balance across brain regions in humans, thus helping us to define the conditions for processing SDMs and contributing to our understanding of DA. Another line of research that could be translated to human patients deals with the potential role of neuronal oscillations in DA. To date, low frequency oscillations have been largely ignored, with most work focusing on theta, alpha, beta and gamma oscillations. However, focusing on delta oscillations could be more relevant for understanding SDM and its role in DA. Lastly, although we only touched on the role of microRNAs as regulators of cellular states, these molecules might show specific profiles in individuals with a history of traumatic stress associated with DA.

In our view, important remaining questions, both for animal and human researchers, relate to the mechanisms by which inaccessible stress-related memories might contribute to psychopathology. If consciously accessible (typically cortically processed) memories of trauma can have debilitating consequences for social behavior, affective behavior, and autonomic function, as they do for PTSD patients, there is no reason to believe that inaccessible memories (typically subcortically processed) would not have similar consequences in DA patients. In fact, one might expect inaccessible memories to have even stronger adverse effects, given the neuroanatomical proximity and connectivity to the amygdala, hypothalamus and other centers for socio-affective and autonomic regulation. Another important issue is under what conditions their successful retrieval might be beneficial or detrimental for patients. Lastly, we know little about extinction of affective (e.g., fear) and behavioral (e.g., avoidance) symptoms related to SDM or DA. More research in this area is needed, particularly in view of the fact that extinction processes themselves are sometimes state-dependent and even facilitated under increased stress levels (Self and Choi, 2004). From a basic science perspective, novel circuit approaches in neurobiology should be able to address these questions by determining the relationship between SDM and affective circuits, in the same way as they have already been applied to research with accessible memories (Ramirez et al., 2015). As already indicated, one of the challenges that we face in investigating these issues experimentally relates to the ethical problems associated with applying extreme stress to animals and humans. In animal research, this can, to some extent be circumvented by working with genetically susceptible individuals that are more likely to engage memory-suppressing mechanisms even when stress is not excessive. In the human domain, we suspect the best approach would be to invest more heavily in genetic, epigenetic, imaging and behavioral studies in patients as a way of providing additional support to existing psychotherapies.

## Revisiting Skepticism Concerning DA

In this final section, we shall make a few observations relating to skepticism surrounding the notion of DA itself (Pope et al., 1998; Piper and Merskey, 2004; McNally, 2007). In doing so, we will focus on issues pertaining to information processing and memory in general. Objections to the idea of DA can be summarized as follows: (1) encoding inaccessible memories is not a “natural capacity” of the brain; (2) there are no recovered memories because the memories in question were never lost or repressed—they were simply forgotten or not thought about in the ordinary way; (3) recovered memories are false memories; (4) there are no recovered memories because there never were any memories to lose or repress in the first place—they were never formed (e.g., due to infantile amnesia); (5) known biological mechanisms of memory show that stress can only enhance memory; and (6) if traumatic stress triggers DA, why is it found only in some traumatized individuals? We should note that whereas we are not convinced by some of the arguments adduced by DA skeptics, we nevertheless agree that raising such questions is a legitimate enterprise especially given the lack of rigorous analyses of DA in early reports.

With respect to objection (1) that encoding inaccessible memories is not a “natural capacity” of the brain (e.g., McNally, 2007), our response is unquestionably that it is, and our conviction is not based only on brain research. In fact, highly restricted state-dependent access to information is a cellular phenomenon, evolutionarily evident as early as in plants (Ku et al., 2015). Under extreme (abiotic) stress, plants completely shift their genetic expression program in such a way as to preclude access to mechanisms that regulate their normal behavior, instead allowing access to mechanisms that give rise to stress-specific adaptive behavior. Moreover, this massive reversal of information processing is regulated by microRNA molecules (Sunkar et al., 2012), which also play a prominent role in neurons and have been implicated in SDM by virtue of regulating GABA receptor levels (Jovasevic et al., 2015). Importantly, brain microRNAs can reach the blood, and can thus contribute to the assessment of processes taking place in the brain. Although more research is needed in this area, the field has already moved significantly towards understanding the relationship between blood microRNAs and psychopathologies such as schizophrenia and depression (Moreau et al., 2011). Similar studies in patients with DA could be helpful as an auxiliary diagnostic tool.

Of the remaining candidate objections two are particularly worth addressing—the “dissociated memories are merely forgotten memories” claim (2), and (3) the “recovered memories are false memories” claim. Defenders of DA as a bone fide condition claim that DA does not follow the rules of ordinary forgetting. In particular, they argue that DA is more likely to occur after repeated episodes, which ordinarily improves remembering, and that unlike normal memory processes, dissociated memories are not sensitive to reminders (Spiegel et al., 2011b). Similarly, Waller et al. (1996) proposed that discontinuity in consciousness associated with DA is an extreme deviation from normality. They proposed that psychopathological dissociation is separate from the normative continuum of dissociation and that rather than a simple cluster of scores at the high end of a continuum, pathological DA is a completely separate construct. This position is taken by others as well (van der Kolk and Fisler, 1995; Harrison et al., 2017), and has received recent support from animal research on SDM. Nevertheless, critics of traumatic amnesia argue that there is nothing special about memories that cannot be accessed during DA (Shobe and Kihlstrom, 1997) and that in fact they represent nothing more than mere forgetting. Unfortunately, the issue of forgetting is difficult to adjudicate because the notions of “normal remembering and forgetting” remain vague. As discussed earlier, the term *forgetting* is ambiguous and quite generic because it can refer to both degraded and inaccessible memories. From a neurobiological perspective, there have been important advances in defining mechanistically different types of forgetting: neurogenesis-based forgetting, interference-based forgetting, and intrinsic forgetting (Davis and Zhong, 2017). However, all of these kinds of forgetting assume partial or complete memory loss. Regarding blocked access forgetting, other difficulties arise because there can be different causes of the memory deficits, including failure of retrieval mechanisms (Ouyang and Thomas, 2005), reactivation-induced memory modifications (Nader et al., 2000; Alberini et al., 2006), or state-dependence (Gisquet-Verrier et al., 2015; Radulovic et al., 2017). In any case, specifying the nature of normal forgetting seems to be essential for developing further the argument on forgetfulness in amnesia.

The objection (3) that recovered traumatic memories are false memories similarly suffers from vagueness as to what it is for a memory to be a false memory. Is the objection the strong, but implausible, claim that “recovered” memories are all complete fabrications, or is it the weaker claim that some (perhaps even many) of the details of such memories are erroneous? As indicated in the section on memory fidelity, there is nothing abnormal about memories being false, especially in the second sense. The problem of false memories is more serious as a societal and legal problem, but from the standpoint of memory research most of our memories are to a certain degree false. Although fragmented memories might indeed render trauma survivors more prone to form trauma-relevant false memories (Jacobs and Nadel, 1998) it is a bit puzzling why this becomes an issue only for memories that were forgotten and then remembered, as in DA, and not for all of our memories, especially given the ease with which false memories can be produced, even in the laboratory (Roediger and McDermott, 1995; Wade et al., 2007). To complicate matters further, in parallel with false memories, another, apparently very robust and quite opposite phenomenon becomes pronounced as we age. This is the “reminiscence bump” whereby in older people early memories start to be over-represented in what they spontaneously recall, resulting in remembering even those early life events to which access has long been denied (Koppel and Rubin, 2016). Thus, rather than taking sides in this debate, it might be more helpful to intensify research in psychology and neurobiology that attempts to differentiate false from veridical memories. A recent study suggests that this might be possible, by demonstrating discrete patterns of brain activity during processing of true, imagined, and high stress-related memories (Palmieri et al., 2014).

We believe that the objection (5) that stress only enhances memory can be rejected on the basis of neuroscientific evidence for memory suppressing effects of stress (recently reviewed, Moreira et al., 2016), and the objection (4) that there can be no recovered memories because such putative memories were never formed in the first place, is an untestable proposition and therefore devoid of empirical content. Neither we, nor the proponents of such a claim, can ever provide any evidence of the non-existence of something.

Finally, Piper and Merskey (2004) have expressed a general concern about the relationship between trauma and dissociative disorders because, among other things, (6) many individuals experience trauma but do not develop the disorder. Although a number of retrospective and prospective studies have identified the role of chronic childhood trauma in the development of dissociative disorders, trauma, although necessary, has never been considered to be sufficient for their emergence (Sanders and Giolas, 1991; Ogawa et al., 1997). We do not find this to be a compelling objection partly because it is now well established that there is no unitary response to traumatic stress. But more important, this kind of wholesale rejection of individual differences is inconsistent with the fact that many genetic, epigenetic and environmental factors confer susceptibility, resilience, or resistance to different psychopathologies.

To sum up, we believe that strong clinical evidence, compelling neurobiological evidence, and well-grounded theoretical arguments all lead to the conclusion that DA is a real phenomenon and that modern advances might enable us to distinguish between legitimate cases of DA on the one hand and contrived cases on the other. Furthermore, we believe that we have provided some convincing reasons for supposing that state dependence constitutes a good explanation of at least some of the mechanisms that underlie DA.

## Author Contributions

JR wrote parts of the overview, the section on neurobiological mechanism and conclusion. RL provided the case study and associated material, and AO wrote substantial parts of the overview and conclusion and the section on cognitive perspective. All authors listed have made a substantial, direct and intellectual contribution to the work, and approved it for publication.

## Conflict of Interest Statement

The authors declare that the research was conducted in the absence of any commercial or financial relationships that could be construed as a potential conflict of interest.
